# Maintenance of Blinding in Clinical Trials and the Implications for Studying Analgesia Using Cannabinoids

**DOI:** 10.1089/can.2016.0016

**Published:** 2016-07-01

**Authors:** Barth Wilsey, Reena Deutsch, Thomas D. Marcotte

**Affiliations:** Department of Psychiatry, Center for Medicinal Cannabis Research, University of California, San Diego, San Diego, California.

**Keywords:** medical marijuana, phytocannabinoids, psychopharmacology

## Abstract

The design of analgesic clinical trials invariably involves a comparison between placebo and active study medication. An assumption is made that treatment effects can be approximated by subtracting the response to placebo from that attained with the use of active study medication. However, the psychoactivity of cannabinoids may unmask their presence and lead to an expectation and/or conditioning of pain relief. For example, study participants biased toward the belief that cannabis is beneficial for their condition might be more inclined to report positive effects if they were to accurately identify the active treatment because of its psychoactivity. This may lead to incorrect assumptions regarding the efficacy of a cannabinoid. Methodologies designed to counteract unmasking need to be implemented in the design phase of a study. During the clinical trial, it is also important to query participants as to which treatment they believe they have received. Blinding can be considered to be preserved when the accuracy of treatment guesses is not considerably different than random guessing, which is estimated to be correct 50% of the time. After a study has been completed, the use of statistical methodologies such as regression and mediation analysis are worthy of consideration to see whether psychoactive effects biased the results.

## Introduction

One of the traditional standards for the conducting of randomized clinical trials (RCTs) is that they be performed double-blind, whereby participants are not able to discern their assigned treatment and researchers are not to be permitted to have knowledge of group assignment so as to avoid influencing their participants' responses. One can then be relatively confident that the distinction between groups derives from therapy rather than the participants' or researchers' biases. However, blinding success (or failure) is rarely reported; in one review, only 31 out of 1599 trials (2%) reported assessments for the success of blinding.^1^

Blinding can particularly be a challenge when the active treatment is difficult to disguise from placebo, as with a psychoactive medication such as cannabis. Recognizable side-effects (e.g., distorted thinking, jocularity, etc.) might lead to poor concealment of group allocation. As a consequence, false conclusions regarding the results of a clinical trial may arise. Compared with trials in which authors reported adequately concealed treatment allocation, trials in which concealment was either inadequate or unclear (did not report or incompletely reported a concealment approach) yielded larger estimates of treatment effects (*p*<0.001).^2^ Odds ratios were exaggerated by 41% for inadequately concealed trials and by 30% for unclearly concealed trials after adjustment for other aspects of quality (e.g., double-blinding, reports on random-number generation, excluding participants after randomization for protocol deviations, withdrawals, dropouts, and losses to follow-up).

Many of the medications (e.g., anticonvulsants used in the treatment of neuropathic pain) studied in recent RCTs have failed to show statistically significant superiority to placebo.^3^ Paradoxically, these studies often involved maladies in which efficacy for these drugs had already been established. The limitation in the ability of these RCTs to demonstrate a benefit of active agent versus placebo illustrates the phenomenon of assay sensitivity, the ability of a clinical trial to differentiate between an effective treatment (e.g., a drug) and a less effective or ineffective treatment (e.g., placebo).^3^ This article discusses the placebo response from the perspective of multiple different types of drugs to improve assay sensitivity in clinical trials involving cannabinergic medications.

## Materials and Methods

The material presented was drawn from influential sources of current and past medical literature. PubMed searches were conducted using the following Mesh Headings: cannabinoids; tetrahydrocannabinol; clinical trial; double-blind method; placebos; and conditioning. Individual articles were selected based on their relevance to the design of future clinical trials involving cannabinoids. As will be discussed later, most of the references were from major medical areas (psychiatry, neurology, and internal medicine) that were known for having a high placebo response rate and not from the cannabinoid literature.

We also briefly report on the results of a survey of participants from a protocol that was part of a larger study that has not yet been published (ClinicalTrials.gov Identifier NCT01555983) and that evaluates the analgesic, neuropsychological, and psychomimetic response to vaporized cannabis in patients with spinal cord injury and disease. The questions asked dealt with whether or not participants believed they were receiving vaporized cannabis or placebo. The results of this survey, as well as other similar surveys, are discussed in the following paragraphs.

## Discussion

### Beliefs about treatment allocation

When cannabinergic medications are studied, investigators should evaluate participants' beliefs about their treatment allocation and attempt to see whether and how these beliefs may have influenced the trial's outcome.^4^ Successful concealment of the identity of cannabis from participants is prone to be challenging because of cannabis' psychoactivity. In fact, reviewers have challenged protocols opining that unblinding of treatment allocation muddles the results of such studies.^5^ Theoretically, participants biased toward the belief that cannabis is beneficial for their condition might be more inclined to report positive effects if they were to accurately identify the active treatment. Insight into this problem comes from outpatient cross-over trials that evaluated maintenance of the blind by asking participants to “guess” assignments at different points of cannabis administration.

In one study,^6^ blinding was considered to be preserved when the accuracy of treatment guesses was no different than random guessing, which was estimated to be correct 50% of the time. After dose titration, participants receiving placebo guessed correctly in 8 of 13 instances (62%), whereas those receiving cannabis guessed correctly 14 out of 15 times (93%). After 2 weeks of washout to allow for drug clearance, 12 of 13 (92%) of the participants crossing over to cannabis from placebo during their second treatment week correctly guessed their treatment assignment.

In another study, participants were randomly assigned to receive cannabis using four potencies: 0%, 2.5%, 6%, and 9.4% delta 9 tetrahydrocannabinol (Δ9-THC) over four 14-day periods in a cross-over trial.^5^ Each period began with 5 days on the study drug followed by a nine-day washout period. On day 5 of the first cycle, 1 of the 5 participants (20%) assigned to placebo correctly identified this assignment, whereas 9 of the 16 participants (56%) who received placebo during later cycles did so. Of the 5 participants administered 9.4% Δ9-THC in their first cycle, none correctly identified this assignment, whereas 10 of the 16 participants (63%) did so during later cycles. At the end of the trial, 16 (76%) of the participants were able to correctly identify the 9.4% Δ9-THC period and 13 (62%) were able to identify the 0% Δ9-THC period; whereas 8 participants identified the 6% Δ9-THC period (38%) and 7 identified the 2.5% period (33%).

In a third study by our group (not yet published), participants were randomly assigned to a sequence of 0%, 2.9%, and 6.7% Δ9-THC administered during three 8-h human laboratory experimental sessions. When combining participant opinions about whether or not active medication was received, 55% of the opinions were correct for placebo, 65% were correct for 2.9% THC, and 93% were correct for 6.7% THC. Opinion accuracy about whether or not treatment was active THC versus placebo was significantly higher for the 6.7% dose than for either placebo (*p*<0.0001, χ^2^(df=1)=15.7) or the 2.9% dose (*p*=0.0022, χ^2^(df=1)=9.4). Accuracy rates for placebo compared with the lower dose were not different (*p*=0.3771, χ^2^(df=1)=0.78).

In the studies mentioned earlier, the accuracy of guessing was linked to two factors. First, participants were more likely to guess what was being provided on subsequent cross-over sessions when they had a prior exposure to placebo or cannabis. Second, higher doses of cannabis also led to greater rates of correct guessing.

### Research designs to improve masking of medications

References to various research designs discussed next are organized for comparative purposes in Table 1.

**Table 1.** **Summary of Research Designs to Improve Masking of Medications**

Author	Study design	Author's conclusions
Gewandter^7,a^	Parallel groups	The randomized, double-blind, parallel group design is the gold standard for confirmatory clinical trials of chronic pain treatments. This design requires larger numbers of participants to detect treatment differences than the cross-over designs.
Gilron^8,a^	Cross-over, active-control trials	Data from a blinding questionnaire indicated that approximately two thirds of the participants guessed correctly regarding receiving placebo. Thus, lorazepam was not successful in preventing unblinding, as the accuracy of correct guesses was greater than random guessing (i.e., estimated to be correct 50% of the time).
Moncrieff^9,b^	Cross-over, active-control trials	The authors advocated active placebos, concluding that unblinding effects may inflate the efficacy of antidepressants in trials when inert placebos are utilized.
Ellenberg^15,b^	Placebo-controlled trials and active-control trials	Prohibition of placebo-controlled trials in settings in which known effective therapy is available would have negative consequences. Trials of new products using active controls would not be able to provide persuasive evidence of efficacy unless the new treatment proved statistically superior to the active control.
Enck^18,b^	Mediating or moderating the placebo response to medicines	In clinical trials, the placebo effect should be minimized to optimize drug–placebo differences. Once the drug is in clinical use, placebo effects should be maximized by harnessing patients' expectations, thus providing a mechanism to improve treatment outcomes.
Fava^23,2^	Sequential parallel comparison design, in which non-responders to placebo are studied in a second phase	This involves two sequential phases of treatment: (1) An unbalanced randomization between placebo and active treatment, with more patients randomized to placebo. (2) Non-responders treated with placebo are randomized to either active treatment or placebo. Since patients in the second phase have already “failed placebo,” their placebo response will be reduced. The analysis pools the data from both phases to maximize power and reduce the required sample size.
Novotna^28,c^	Enriched enrolment design, in which responders to active treatment are studied in a second phase	The enriched study design provides a method of determining the efficacy and safety of medications in a manner that more closely reflects clinical practice, by limiting exposure to those patients who are likely to benefit from it. Consequently, the difference between active and placebo should be a reflection of efficacy and safety in the population intended for treatment.
Weimer^29,b^	Meta-analysis of study designs and placebo responses	Higher placebo responses are found with: • greater symptom severity at baseline • more recently performed studies than studies in the past • more study visits during the trial and studies • randomizing more patients to drug than to placebo (unbalanced randomization)
Katz^31,a^	Identify factors associated with positive (i.e., favors medication) versus negative outcomes of placebo-controlled neuropathic pain trials	Requiring higher baseline pain (e.g., 5 or 6 on a 0 to 10 numerical rating scale rather than 4 or below) in neuropathic pain trials might make positive outcomes more likely.
Khan^32,b^	Study designs and outcomes in antidepressant clinical trials	The two most notable factors affecting positive trials are (1) the inclusion of patients with more severe depression and (2) the use of a flexible-dose design; these may yield results, identifying true antidepressant-placebo differences
Quitkin^34,b^	Extending duration of study	Length of treatment may affect results. In some studies, the proportion of patients showing a clear-cut response increased significantly among patients treated with active drug instead of placebo when the treatment period was extended from 4 to 6 weeks, independent of the dose used. There may, thus, be a distinct advantage in extending trials of antidepressants for a minimum of 6 weeks. Twelve-week trials might increase the statistical power of the evaluation by 10–20%, in studies where the drug effect size is small.
Potkin^35,b^	Extending duration of study	Long-term (longer than 1 year), randomized, double-blind, placebo-controlled trials may still have high placebo response rates.
Khan^32,b^	Flexible dose design	Flexible dose research designs were almost twice as likely to demonstrate significant differences between antidepressants and placebo as fixed-dose trials.

^a^ Study involves chronic pain treatments.

^b^ Major medical areas (psychiatry, neurology, and internal medicine) known for high placebo response rates.

^c^ Involves cannabinoid treatments.

#### Using an active placebo in cross-over studies

Compared with cross-over trials, parallel-group designs might obviate some of the concerns regarding unblinding. But such designs often require considerably greater sample sizes, financial resources, and investment of personnel, making it difficult, if not impossible, for many investigators to use this type of clinical trial.^7^ Therefore, it is appropriate to discuss mechanisms by which cross-over trials might improve assay sensitivity. In this regard, one might consider the use of an active placebo. Presumably because of the difficulty in matching an active placebo to the treatment under consideration, there have not been that many trials using this method. One study, however, assessed the effects of morphine and gabapentin on neuropathic pain using lorazepam as the active placebo.^8^ Although not analgesic, lorazepam was considered appropriate, because it triggers similar side-effects to morphine (e.g., sleepiness and dizziness). In this randomized, double-blind, active, placebo-controlled, four-period cross-over trial, participants received daily active placebo (lorazepam), sustained-release morphine, gabapentin, and a combination of gabapentin and morphine, each given orally for 5 weeks. The trial unequivocally demonstrated that gabapentin significantly enhanced the efficacy of morphine. However, data from a blinding questionnaire indicated that approximately two thirds of the participants guessed correctly regarding receiving placebo. Thus, lorazepam was not successful in preventing unblinding, as the accuracy of correct guesses was greater than random guessing (i.e., estimated to be correct 50% of the time).

Other researchers have offered more favorable opinions related to active placebos. A meta-analysis of nine studies involving 751 participants reviewed the efficacy of tricyclic antidepressants when compared with active placebos (primarily atropine; phenobarbital was used once).^9^ Only one study used a cross-over design; the others utilized parallel groups. Combining all studies produced a pooled estimate of effect of 0.39 standard deviations (confidence interval 0.24–0.54) in favor of the antidepressant, as measured by improvement in mood. The authors advocated active placebos, concluding that unblinding effects may inflate the efficacy of antidepressants in trials when inert placebos are utilized.^9^

Insofar as studies with cannabinoids are concerned, the utility of an active placebo was demonstrated in a randomized, double-blind, active-control, equivalency cross-over trial that compared nabilone (0.5–1.0 mg before bedtime) with amitriptyline (10–20 mg before bedtime) in patients with fibromyalgia with chronic insomnia.^10^ Because both drugs cause similar side-effects (e.g., drowsiness and dry mouth), the authors postulated that amitriptyline would be a suitable active control for nabilone, and, therefore, would preserve masking. When asked at the end of the study to guess which treatment had been administered, 8 subjects (29%) correctly identified the period in which they received amitriptyline, and 12 (41%) correctly identified the period in which they received nabilone. Blinding was considered to be preserved, as the accuracy of guesstimates was less than 50%, a level consistent with random guessing. This suggests that amitriptyline would be a good active control for trials involving cannabinoids. In considering the options for future trials, the side-effect profiles of lorazepam, atropine, phenobarbital, amitriptyline, and cannabis are presented in Table 2. Hypothetically, lorazepam would be the optimal active placebo in a cannabis trial, as the two medications share many side-effects (e.g., euphoria, drowsiness, dizziness, trouble concentrating, etc.). The failure of lorazepam to not prevent unblinding in the study mentioned earlier involving morphine and gabapentin might have been secondary to insufficient dosing. The daily dose of what was referred to as “low-dose lorazepam” in that study was 1.6 mg in divided (0.1 or 0.2 mg) doses. Perhaps a higher dose might have had more success at masking than the dose selected. The usual range in clinical practice is 2 to 6 mg/day.^11^ Of course, over-sedation might be a potential problem. In an attempt to avoid this, one would exclude chronic pain patients taking opioids and/or other benzodiazepines concurrently.^12^ In addition, operating heavy machinery and/or driving a motor vehicle would not be permitted for 3 to 4 h after dosing of study medication. As the literature supports advising participants to refrain from these activities for this period after the intake of cannabinoids,^13^ a clinical trial comparing a cannabinoid with lorazepam would already have this safety feature operative.

**Table 2.** **Side-Effect Profile of Cannabis Compared with Potential Active Placebos**

Cannabis side-effects^59^	Lorazepam^60^	Phenobarbital^61^	Amitriptyline^62^	Atropine^63^
ENT				
Dry mouth			+	+
Cardiac				
Tachycardia				+
GI				
Nausea	+	+		
Vomiting	+	+		
Neurological				
Drowsiness	+	+	+	
Dizziness	+	+	+	
Forgetfulness	+			
Trouble concentrating	+			
Headache	+	+		
Psychiatric				
Hallucinations	+	+		
Depression	+	+		
Erectile dysfunction	+			
Euphoria	+			

Similar side-effects to those of cannabis are symbolized with a plus sign beneath the listing of each medication.

#### Omitting the placebo

The Declaration of Helsinki, the World Medical Association's best-known policy statement, upholds an ethical standard whereby all participants are to receive “the best treatment” available. At odds with this are trials that include a placebo arm, whereby one withholds effective treatment for scientific purposes.^14,15^ The first version of the Declaration was written and adopted in 1964; this document has been amended seven times in the intervening years, most recently at the General Assembly in October 2013.^16^ According to the latest version, the benefits, risks, burdens, and effectiveness of a new intervention must be tested against those of the best proven intervention(s), except in the following circumstances:

• Where no proven intervention exists
• Where for compelling and scientifically sound methodological reasons, the use of any intervention less effective than the best proven one may be used.

The revisions recognized the obstacle to the scientific evaluation of a number of drugs and treatments. In one critique of the Declaration of Helsinki, the argument was made that the use of placebo is justified whenever its use does not cause irreversible damage or considerable suffering to the well-informed participant.^17^ In addition, the argument was put forward that a placebo control is of value in those diseases with a tendency toward spontaneous improvement or in those with a pronounced psychological component.^17^ The latter line of reasoning would certainly be applicable to analgesic research.

#### Comparative effectiveness research

The dilemma of not using a placebo is avoided in comparative effectiveness research (CER), which delivers active treatment to all participants.^18^ CER trials are a particular focus for the National Institutes of Health.^19^ Such trials compare innovative compounds with approved drugs or standard therapy. Unfortunately, a finding of no difference in an active-control study can mean that both agents are effective, that neither is effective, or that the study was simply unable to tell effective from ineffective agents.^20^ This dilemma arises out of the assumption that knowledge that the active control is superior to a placebo may not be verifiable.^20^ Failure of placebo-controlled trials is not uncommon; new analgesics and antidepressants often fail to show superiority to placebo.^21^ Therefore, comparative effectiveness trials that demonstrate no difference between the new drug and active control may be of little value.^20^ There is another problem with CER. Dependent on the relative effect sizes, more participants may be needed in this type of drug trial if the difference between active treatments is smaller than that between active treatment and placebo. This is in conflict with another tenet of the Declaration of Helsinki, whereby the minimum number of participants should be exposed to drug testing.^22^

#### Enriched enrollment

As with the use of an active placebo, other suggestions have been made to alter the research design to reduce or eliminate the placebo effect.^22^ The sequential parallel comparison (SPC) design was proposed to improve the efficiency of psychiatric clinical trials by reducing the impact of placebo response.^23,24^ This strategy involves two consecutive placebo-controlled comparisons, of which the second is entered only by placebo non-responders from the first phase. At the completion of phase I, participants are designated responders or non-responders based on pre-determined study criteria. Placebo responders are asked to withdraw from the double-blind study altogether or, if part of the study design, may continue with open-label therapy. Placebo non-responders enter phase II and are randomized to active drug or placebo. Previous studies suggest that in antidepressant trials, nonresponse to placebo can already be predicted after 2 weeks of follow-up.^25^ This would allow a reduction in the time spent in the study by participants in the first phase of the SPC design and, thus, an increase in its efficiency. But exclusion of placebo responders has not had the success that might be expected from such a maneuver.^26^ For example, despite the use of the SPC, placebo response rates still hovered at unacceptable levels between 30% and 40%.^27,28^ Although attempts to decrease the placebo response in antidepressant clinical trials have generally not been effective,^21^ it is not known whether this technique would be effective in cannabinoid clinical trials.

The corollary to reducing placebo responders is to increase the number of participants who respond to active medication. This type of design involving responders has had some success in a cannabinoid study involving Sativex^®^, an oromucosal spray containing Δ9-THC and cannabidiol (CBD) in a 1:1 ratio. This study employed an enriched design in which two phases were conducted.^29^ Only those participants who showed an initial response during a preliminary 4-week single-blind phase (phase A), defined as a ≥20% reduction in the spasticity 0–10 numerical rating scale score, were eligible for randomization in the subsequent 12-week double-blind phase (phase B) of the study. Of the 572 participants who entered phase A across 51 study sites, 241 were initial responders and met the criteria to progress to phase B. After 12 weeks' treatment, the intention-to-treat analysis showed a significant difference in favor of Δ9-THC:CBD oromucosal spray over placebo (*p*=0.0002). It could be argued that this is stacking the deck to find a positive result. On the other hand, a case could be made for clinical trials that show an evolution toward personalized medicine, whereby those individuals who are the most likely to benefit from specific treatment are identified. Subsequently, one might find correlates, that is, genetic markers, in those participants who respond to a specific treatment that could then be used in a clinical setting.

#### Selecting participants with increased illness severity

Another suggestion that has been made is to alter the research design to enroll participants with greater symptom severity at baseline to influence the placebo response.^30^ As an example, the results of an exploration of three clinical trials of painful diabetic neuropathy indicated that the difference between active medication and placebo was larger in participants with greater baseline pain intensity.^31^ As a result, it has been suggested that inclusion criteria requiring higher baseline pain (e.g., 5 or 6 on a 0 to 10 numerical rating scale rather than 4 or below) in neuropathic pain trials might make positive outcomes more likely.^32^ In an analogous manner, selection of participants with more severe depression was believed to yield results that identified true antidepressant-placebo differences. In one study, severely ill participants with depression responded well to antidepressants but poorly to placebo.^33^ Unfortunately, attempts to restrict clinical trials to populations with greater illness severity have typically failed.^23^ There is no experience with this design using cannabinoids, but the prospect of using participants with a higher baseline pain would seem to be a feasible goal in the future.

#### Extending the duration of study

Extending the trial duration has also been believed to decrease the placebo response. This followed from early observations that specific antidepressant drug effects most likely occur after the first 2 weeks of treatment and are stable, whereas placebo responses tend to appear early and have variable duration.^34,35^ As a result, many investigators advocated study designs where the duration of the double-blind trial was extended to 8 or 12 weeks for major depressive disorders, and for even longer periods for some of the other psychiatric disorders.^23^ Whether this tactic has led to any reduction in the rate of the placebo response remains questionable and, perhaps, unwise in its propensity to burden participants and investigators unnecessarily. For instance, it has been reported that long-term (longer than 1 year), randomized, double-blind, placebo-controlled trials may still have high placebo response rates.^36^ A significant concern, of course, is the potential risk to participants who may need treatment and end up in a long-term period of placebo treatment. Participant safety would, thus, be jeopardized unnecessarily if the study outcome could adequately be determined via a shorter trial.

#### Flexible dose design

The option of flexible dose design, in which the participant balances benefits and side-effects by selecting a dosage among various options, appears to be more promising than several of the aforementioned design changes. The results of a meta-analysis indicated that trials with flexible dose research designs were almost twice as likely to demonstrate significant differences between antidepressants and placebo as fixed-dose trials.^33^ Higher placebo response rates in the fixed-dose trials might be explained by an increase in expectations of receiving a beneficial treatment. In this scenario, the participant believes the “doctor-knows-best” and has optimized the treatment regimen. This is similar to the situation in clinical practice in which the clinician adjusts doses to optimal efficacy and tolerability according to individual patient requirements. In the case of a flexible dose design, a presumption might be made by the participant of a lack of medical knowledge theoretically reducing (or at least not increasing) expectations. This methodology has been previously accomplished in a cannabinoid study involving the treatment of neuropathic pain with Sativex, where participants self-titrated their overall dose and pattern of dosing according to their response to and tolerance of the medicine.^37^

### Evaluation of blinding

#### Preparations to monitor blinding before the study

The importance of blinding has been emphasized by the CONSORT group.^38^ Theoretically, a description of the success of blinding in a formal manner may become a requirement for publication in the future. Even without mandatory reporting, drug trials that did not measure blinding miss an opportunity to assess a significant factor that drives placebo effects. Understandably, such monitoring must be planned before study implementation.^39–41^ The most frequently used approach is querying participants as to their guess of which treatment they believe they have received, and to assess the statistical significance of the results. However, there is no established methodology on how to ask the question or on how to analyze the results.^1,42^ The statistical procedure used to assess the significance of the guesses differs among studies, with a Chi-square or Fisher's exact test being the most commonly employed.^42^ A kappa statistic has also been used commonly to measure the degree of agreement between the guess and the true assignment.

A more sophisticated measure is the blinding index, which provides a more systematic way of dealing with inferences regarding treatment allocation by including not only the presumption of assignment but also a “don't know” option.^43^ The blinding index is a variation of the kappa statistic; however, although the standard kappa statistic ignores “don't know” responses and measures the degree of agreement, not knowing is a more favorable result, since it indicates a high degree of blinding. This instrument is scaled on an interval of 0 to 1, with 0 being complete lack of blinding and 1 being complete blinding.

As such, objectively calculated blinding data may offer meaningful and systematic ways to further interpret the findings of randomized, controlled trials, and blinding has even been studied in meta-analysis.^44,45^ This has been done for acupuncture trials in which little agreement exists among researchers as to how to control for insertion of an acupuncture needle. Furthering the literature on this subject, the effectiveness of blinding was shown to be satisfactory in one meta-analysis, with 61% of study participants maintaining ideal blinding.^45^

It is known that the active medication and placebo arms of a clinical trial can manifest different degrees of blinding.^46^ As the blinding index is a single value that combines blinding data from all study arms, it does not make this distinction. A modified blinding index was, therefore, devised to address this issue.^47^ This revision incorporates the ability to detect different behaviors in different treatment arms, including the “wishful thinking” scenario in which trial participants tend to think they are allocated to the experimental group, even if not in reality.^48^ It provides a value between −1 and 1, with 0 as a null value, which indicates the most desirable situation under random blinding. A positive value may imply failure in blinding above random guessing (i.e., a majority of participants guess their treatment allocation correctly), and a negative value may suggest the success of blinding or failure of blinding in the other direction (i.e., more individuals mistakenly name the alternative treatment).

With the advent of blinding indices, the questions asked of research participants have become more sophisticated. One proposed method is to ask participants “Which treatment do you think you received (or were assigned to)?” and to give them the following choices: (1) Treatment A; (2) Treatment B; and (3) Don't know. More varied choices are (1) Strongly treatment A; (2) Somewhat treatment A; (3) Don't know; (4) Somewhat treatment B; and (5) Strongly treatment B.^49^ An important supposition in either blinding index is that when a respondent says that she or he does not know the identity of the agent, this represents a sincere response, not one that simply evades making a judgment.^46^ In this regard, it is important that the investigative team urges respondents to report their thoughts honestly when questions about the identity of a treatment exist.

The optimal timing and frequency of blinding assessments has not been established.^46,49^ Although expectations of the participants are not measured directly by the blinding index, responses at an early stage of the trial might reflect a person's preference or wish that they are receiving active treatment. Consequently, it has been recommended that blinding be assessed at the end of the trial, at which time the results will summarize the overall maintenance of successful masking.^48^ On the other hand, end-of-trial blindness assessments might actually be a test for recognition of side-effects rather than a measurement on the success of blinding. End-of-trial tests of blindness might also represent an assessment of efficacy whereby participants recognize a predictable effect. Under these assumptions, it is recommended that the success of blinding preferably be assessed in the early stages of the trial before the presentation of side-effects or demonstration of efficacy.^50–52^ One strategy would be to collect blinding data two times from participants: shortly after randomization (at the clinic) and at the end of the trial (e.g., by phone).^49^ This would permit a comparison of blinding at the two stages.

#### Describing blinding after the study

Investigations of blinding after a trial has completed enrollment and is in the analysis phase may be performed analytically. For example, in one study,^53^ the mechanisms of the self-reported primary outcome measure, visual analog scale pain intensity, were further evaluated by adding psychomimetic effects of cannabis (e.g., feeling stoned, high, drunk) as covariates to a mixed-model regression to determine whether there was a reduction or an elimination of the analgesic effects of cannabis by these side-effects. In this instance, the effect of the cannabis treatment maintained significance (all *p*<0.0001) above and beyond any influence of the fifteen different side-effects measured in this study.

A similar technique was employed using efficacy data from three randomized, parallel-group, double-blind trials comparing Sativex with placebo. In these instances, the relationship between factors that might permit participants to identify their therapy allocation and the effect of treatment on the self-reported primary outcome measure, spasticity severity, was investigated. A general linear model was devised, where the dependent variable was the change from baseline in participant self-reported spasticity severity, whereas the various possible explanatory factors (e.g., dizziness, disturbance in attention, euphoric mood, difficulty with speaking, and other variables) were regarded as fixed factors in the model.^54^ There was no significant relationship between the effect of Sativex on spasticity and the prior use of cannabis or the incidence of “typical” adverse events of cannabinoids. The authors concluded that there is no evidence to suggest that there was widespread unblinding to treatment allocation in the three studies examined.

Another method to test whether a treatment effect may be linked to cannabis' side-effects would be to use statistical mediation models, allowing for multiple mediators to be tested simultaneously.^55–57^ The goal would be to empirically quantify and test hypotheses about the contingent nature of the mechanisms by which X exerts its influence on Y.^57^ Such an analysis could be used to establish the extent or pathway to which side-effects influence pain relief. In this scenario, pain relief might be impacted by previous use of cannabis, whereby psychomimetic or neuropsychological effects are recognized. Theoretically, this could arise from classical conditioning if the participant were not cannabis naïve and has had pain relief accompanied by side-effects from cannabis earlier. The analysis might be able to be accomplished by piecing together parameter estimates from a mediation analysis with parameter estimates from a moderation analysis and combining these estimates in ways that quantify the conditionality of various paths of influence from X to Y.^57^

## Conclusions

Maintenance of the blind-to-treatment allocation is one of the most important means of avoiding bias in randomized, controlled clinical trials^54^; however, blinding success is rarely examined and reported. Commonly used methodologies to determine whether participants have become unblinded to treatment allocation are imperfect, particularly in studies where self-reported outcomes are utilized and with medications that have recognizable side-effects. Increased attention to the methodological aspects of research methods have the potential to enhance assay sensitivity in such clinical trials. Attributes that hold promise for reducing the placebo effect include: use of an active placebo, CER where the comparator is known to be superior to placebo, increasing the number of participants who respond to active medication, and flexible dose designs.

To assess blinding, it is necessary to query research participants as to whether or not they believe they received active study medication or placebo. Because it may be misleading to only consider the frequency of correct suspicions as successful or unsuccessful blinding, a “don't know” guessing option would be a valuable adjunct. In addition, the use of statistical methodologies are worthy of consideration during the analysis. Because regression and mediation analysis can be performed after a study is completed, journal reviewers can request that this be performed if there is a suspicion that blinding was not carried out effectively.

Predictors of the placebo response and effective methods to measure and reduce its effect are still to be uncovered. We are early in the process of appraising masking of psychoactive medications, and much work remains to be done.

## Abbreviations Used

CBD: cannabidiol
CER: comparative effectiveness research
Δ9-THC: delta 9 tetrahydrocannabinol
RCTs: randomized clinical trials
SPC: sequential parallel comparison
